# Multiple Morphometric Assessment of Microglial Cells in Deafferented Spinal Trigeminal Nucleus

**DOI:** 10.3389/fnana.2019.00103

**Published:** 2020-01-22

**Authors:** Nuria García-Magro, Yasmina B. Martin, Alejandra Palomino-Antolin, Javier Egea, Pilar Negredo, Carlos Avendaño

**Affiliations:** ^1^Department of Anatomy, Histology and Neuroscience, Medical School, Autonomous University of Madrid, Madrid, Spain; ^2^Ph.D. Programme in Neuroscience, Doctoral School, Autonomous University of Madrid, Madrid, Spain; ^3^Department of Anatomy, Faculty of Medicine, Francisco de Vitoria University, Madrid, Spain; ^4^Research Unit, Hospital Universitario Santa Cristina, Instituto de Investigación Sanitaria Hospital Universitario de La Princesa, Madrid, Spain

**Keywords:** microgliosis, dorsal horn, Iba-1, CD11b, CD45, CD206, stereology, flow cytometry

## Abstract

Microglia (MG) are the first cells to react to the abnormal incoming signals that follow an injury of sensory nerves and play a critical role in the development and maintenance of neuropathic pain, a common sequel of nerve injuries. Here we present population data on cell number, soma size, and length of processes of MG in the caudal division of the spinal trigeminal nucleus (Sp5C) in control mice and at the peak of microgliosis (7 days) following unilateral transection of the infraorbital nerve (IoN). The study is performed combining several bias- and assumption-free imaging and stereological approaches with different immunolabeling procedures, with the objective of tackling some hard problems that often hinder proper execution of MG morphometric studies. Our approach may easily be applied to low-density MG populations, but also works, with limited biases, in territories where MG cell bodies and processes form dense meshworks. In controls, and contralaterally to the deafferented side, MG cell body size and shape and branching pattern matched well the descriptions of “resting” or “surveillant” MG described elsewhere, with only moderate intersubject variability. On the superficial laminae of the deafferented side, however, MG displayed on average larger somata and remarkable diversity in shape. The number of cells and the length of MG processes per mm^3^ increased 5 and 2.5 times, respectively, indicating a net 50% decrease in the mean length of processes per cell. By using specific immunolabeling and cell sorting of vascular macrophages, we found only a negligible fraction of these cells in Sp5C, with no differences between controls and deafferented animals, suggesting that blood-borne monocytes play at most a very limited role in the microgliosis occurring following sensory nerve deafferentation. In sum, here we present reliable morphometric data on MG in control and deafferented trigeminal nuclei using efficient methods that we propose may equally be applied to any morphometric population analysis of these cells under different physiological or pathological conditions.

## Introduction

In the central nervous system, the microglia (MG) were classically assigned a function as sensors of damage and inflammatory and neurodegenerative pathological events (Kreutzberg, 1996), although more recently additional key roles were demonstrated in a number of physiological events, particularly related to the shaping of connections that hallmarks development and plasticity (Kettenmann et al., 2013; Wolf et al., 2017). In response to appropriate stimuli, MG cells swiftly respond by proliferating and shifting their phenotype from “resting” or “surveillant” to various forms of “activated” states. This reaction, initially interpreted in connection with removing cellular debris and promoting neuronal apoptosis and recovery, includes a cohort of changes in cell number, morphology, gene expression, cytokines release, and antigenic profile presentation (Avendaño, 1983; Bruce-Keller, 1999; Streit et al., 1999; Hanisch and Kettenmann, 2007).

While stimuli triggering MG activation must occur in the central nervous system, their origin may be elsewhere. Thus, when a peripheral sensory nerve is damaged, a prominent accumulation of MG in a reactive state, or “microgliosis” for short (Hanisch and Kettenmann, 2007), develops in the spinal and brain stem territories where primary afferents distribute (Gilmore and Skinner, 1979; Cova et al., 1988; Gehrmann et al., 1991; Eriksson et al., 1993; Melzer et al., 1997). MG are the first cells to react locally to signals derived from the affected afferent axons and their cellular targets (Kettenmann et al., 2011), initiating a cascade of events fundamental for the development and maintenance of neuropathic pain (Tsuda et al., 2003; Inoue and Tsuda, 2009; Calvo and Bennett, 2012), a common sequel of nerve injuries. The need to elucidate the cellular and molecular features of MG to understand their diverse roles is undisputed, as is the importance of advancing current knowledge of the variegated MG signatures to enable designing more effective treatments of chronic pain and other disorders (Tsuda et al., 2005; Hanisch and Kettenmann, 2007; Echeverry et al., 2008; Zhuo et al., 2011; Butovsky and Weiner, 2018; Kohno et al., 2018). Nevertheless, the characterization of “activated” MG is a challenging task for several reasons: In the first place, the molecular phenotype and gene expression of MG show remarkable age-, sex-, and region-dependent heterogeneities (Jeong et al., 2016; De Biase et al., 2017; Guneykaya et al., 2018; Masuda et al., 2019), which makes it unadvisable to extrapolate data from one setting to another without due validation. Also, MG respond to damage or pathology in idiosyncratic and context-dependent manners (Hammond et al., 2019; Masuda et al., 2019), probably by selecting clones that eventually are down-regulated in the resolution phase of the disorder (Tay et al., 2017). And for any given damage affecting a specific region time must be factored in since gene expression may change substantially over the course of the lesion or dysfunction. For example, following spinal nerve transection that led to neuropathic pain, spinal MG displayed marked changes in their gene profile along the first postlesion week, and the analysis of differentially expressed genes indicated differential enrichment of some functions or signaling pathways at the initiation of the process or when allodynia was fully expressed (Jeong et al., 2016).

Because of these complexities, it is now widely held that morphology alone is insufficient to distinguish among all possible functional states of MG, let alone when these cells react to lesion or pathology (Jeong et al., 2016; Hirbec et al., 2017). On the other hand, changes in number, size, and shape are stereotypical features of MG “activation” in general, and particularly in all cases of experimental models of nerve injury (Beggs and Salter, 2007; Calvo and Bennett, 2012). Unsurprisingly, morphometry continues to contribute key parameters to assess the dynamics of MG and gain insights into its function (Tremblay et al., 2010; Kettenmann et al., 2011; Beynon and Walker, 2012; Morrison and Filosa, 2013; Parada et al., 2015; Ohgomori et al., 2016; Leon-Espinosa et al., 2018). However, an optimal morphometric approach that yields unbiased results with functional significance and growing efficiency is yet to be found. This justifies the frequent proposal of new approaches, or revisiting of older ones (Yamada and Jinno, 2013; Plog et al., 2014; Torres-Platas et al., 2014; Fernandez-Arjona et al., 2017; Morrison et al., 2017; Young and Morrison, 2018; Chiu et al., 2019).

In this work we present population data on cell number, soma size, and length of processes of MG in the caudal division of the spinal trigeminal nucleus (Sp5C) in control mice and at the peak of microgliosis following unilateral transection of the infraorbital nerve (IoN). The study is performed combining an efficient multiple imaging and stereological approach with different immunolabeling procedures. In addition to providing new data on a scarcely investigated issue (Eriksson et al., 1993; Melzer et al., 1997; Piao et al., 2006), here we tackle some hard problems that often create measuring biases in morphometric studies of MG. Moreover, we present data that support that blood-borne monocytes play at most a very limited role in the microgliosis that follows sensory nerve deafferentation.

## Materials and Methods

### Experimental Subjects and Surgery

Young adult (2–3 months old) male C57BL/6 mice (*n* = 19) were used. All animal procedures were approved in advance by the Ethical Committee of the Autonoma University of Madrid in accordance with the European Community’s Council Directive 2010/63/UE. Appropriate actions were taken to minimize the suffering of the animals and to keep the number of animals used to the minimum that was expected to provide reliable results. Twelve animals underwent irreversible unilateral deafferentation of the trigeminal nuclear complex by unilateral transection and ligation of the right IoN (group IoN). Surgery was performed under an i.p. anesthesia with a mixture of ketamine (0.075 mg/g)–xylazine (0.02 mg/g), and the proximal and distal stumps of the nerve were ligated with silk sutures. Another seven animals were left intact (group C). Seven days later, six deafferented mice were deeply anesthetized (Dolethal, 50 mg/kg i.p.) and perfused through the ascending aorta with 0.9% NaCl (50 ml, 2 min) followed by 4% paraformaldehyde in 0.1 M phosphate buffer (PB; pH 7.4, 200 ml, 10 min, 10–12°C). The brain stem was extracted, and a block containing at least the rostral two-third of the Sp5C nucleus (including the first cervical spinal segment and the medulla up to the obex) (Garcia-Magro et al., 2018) was removed, postfixed in the same fixative overnight, and subsequently cryoprotected for 2 days in 30% sucrose in PB. Three of the control animals were likewise treated in parallel.

The remaining 10 animals (six IoN-transected a week before, and four controls) were likewise anesthetized and briefly perfused (20–30 s) through the heart with sterile saline. A block containing the caudal brain stem and upper spinal cord was quickly exposed and removed, and the dorsolateral quadrant, putatively containing our target region, was carefully dissected out on a cold plate under a stereomicroscope. Both sides of all controls were pooled, as were the left sides of all transected mice and, separately, the deafferented (right) sides of the transected animals.

### Tissue Preparation and Immunostaining

The blocks were frozen and serially cut at 40 μm in the coronal plane in a sliding microtome. Every fifth section was used for free-floating immunostaining. The first series of sections was incubated for two nights at 4°C with a combination of two primary antibodies, rabbit anti-Iba1 (1:500; Wako), and mouse anti-NeuN (1:100; Abcam). After several washes with saline PB (PBS), the sections were incubated for 2 h in the dark in a mixture of secondary antibodies: AlexaFluor 488 donkey-anti-rabbit and AlexaFluor 546 donkey-anti-mouse. In addition, all nuclei were labeled with Bisbenzimide (Hoescht).

The second series was processed using the avidin–biotin–peroxidase (ABC) method with diaminobenzidine (DAB) as a substrate (Fernández-Montoya et al., 2018). Briefly, after several washes in PBS, inactivation of endogenous peroxidase with 1% H_2_O_2_ in PB and preincubation in a blocking solution with 2% Triton X-100 for 1 h, the sections were incubated with rabbit anti-Iba-1 (1:500; Wako) overnight at 4°C. Biotinylated goat anti-rabbit (1:500; Sigma-Aldrich) was used as secondary antibody. Finally, the sections were incubated in avidin–biotin (ABC Elite^®^ Kit, Vector Laboratories) in 0.02 M PBS with 2% Triton X-100 and developed in 0.05% DAB in 0.1 M PB with 0.008% cobalt chloride and 0.0064% nickel sulfate adding 0.001% H_2_O_2_. Sections were then mounted on glass slides, dehydrated, defatted, and coverslipped with DePeX.

In two cases with IoN transection a third series of sections was processed for immunofluorescence as above but replacing the anti-NeuN primary antibody with rat anti-CD206 (1:100; BioRad).

### Three-Dimensional Morphometric Analysis With Imaris

Confocal 3D images were acquired using the z-stack function of a LSM 700 confocal microscopy (Zeiss, Oberkochen, Germany). Images were collected at 1 μm intervals with a 40× oil-immersion objective and a resolution of 2048 × 2048 pixels. Morphological analysis was performed on 3D images using Imaris 7.6.4 software (Bitplane, Zurich, Switzerland). Twenty-nine MG cells with the cytoplasm in laminae I-II of Sp5C and approximately placed at mid-depth of the tissue section and far from the tissue edge were selected for analysis. Their 3D reconstruction was performed using Imaris’ Filament Tracer with no loops allowed and spot detection mode to determine start and end points. Although the analysis was performed automatically by the software, we separately verified that each process originated in a defined cell and manually removed false connections. Among the various morphometric parameters that Imaris provides, just two (number of primary process and total process length) were analyzed in detail that enable comparisons to be made with the same parameters obtained by other methods (see below).

### Quantitative Analysis of Microglia on DAB-Reacted Immunostained Sections

The density of MG cell bodies (*N*_*V*_) and the length density of MG processes (*L*_*V*_) were estimated by means of the optical disector method (Sterio, 1984) and the global spatial sampling procedure of the isotropic virtual planes (Larsen et al., 1998), respectively. The average length of processes per MG cell (*L*_*N*_) was obtained by dividing *L*_*V*_ by *N*_*V*_. All measurements were performed in an integrated stereological setup that included a BX61 Olympus microscope with a high-precision motorized microscope stage (Prior Proscan II, Prior Scientific Inc., Rockland, MA, United States), a 0.1 μm resolution *z*-axis encoder, and an Olympus DP71 high-resolution video camera (Olympus-Europa, Hamburg, Germany). The interactive test grids and the control of the motorized specimen stage were provided by the NewCAST stereological software package (Visiopharm, Hørsholm, Denmark).

To generate virtual planes, the program systematically selects a new isotropic random orientation for all planes that may appear on each new field of vision. An arbitrary distance *d* between planes was set here at 20 μm. A sampling box with fixed *x*, *y*, and *z* dimensions was defined, ensuring that guard areas remained along the three axes outside the box. By keeping constant *d* and the box volume, it was ensured that the sampling density was constant. An isotropic virtual plane is visualized as a line isotropically oriented in 2D (*x*,*y*) that “moves” sidewise when focusing at a “speed” that varies depending on the tilt of the plane with respect to the focusing direction. This tilt contributes isotropy along the *z*-axis.

The target region was defined as the laminae I–II of the ventral one-half of the Sp5C nucleus, representing the caudalmost territory innervated by C and Aδ fibers from the IoN (Garcia-Magro et al., 2018). The region was outlined under a planachromatic 4× dry lens using the drawing tool of NewCAST on five to six sections at 200 μm intervals. Sampling boxes were then systematically placed covering 10% of the target region using a planapochromatic 100× oil-immersion lens (Olympus UPLSAPO, NA = 1.4). The intersections of the test lines with immunostained MG processes were counted applying Larsen et al. (1998) counting rules and their estimator (Eq. 1) to estimate the global length density of the processes as:

$${\hat{L}}_{v}=2\cdot \frac{\Sigma \,Q}{\Sigma \,a\,\left(\mathrm{plane}\right)}=\frac{2\cdot p\,\left(\mathrm{box}\right)}{a\,\left(\mathrm{plane}\right)}\cdot \frac{\Sigma \,Q}{\Sigma \,p\,\left(\mathrm{ref}\right)}$$

where Σ*Q* is the total sum of intersections counted, Σ*a*(plane) is the total area covered by the sampling planes, *p*(box) is the number of points used that represent the sampling box (here, the four topmost corners), *a*(plane) is the area of the sampling planes within each box, and Σ*p*(ref) is the total sum of box corners that hit the reference space.

Section thickness *t* was measured at every third sampling spot by up-and-down focusing. The average Σ*Q*-weighted *t* (Bermejo et al., 2003) of each section was used to correct the sampled volume estimates for variations of shrinkage along the *z*-axis.

The numerical density (*N*_*V*_) of MG cell bodies in the target region was estimated by using optical disector probes (Sterio, 1984) on the same sections used to estimate *L*_*V*_ and was also corrected for vertical shrinkage. Because of the relative scarcity of these cells (at least in control cases), sampling intensity across sections was quadrupled to 40%. The average length of processes per MG cell was then computed as *L_*N*_* = *L_*V*_/N_*V*_*.

The precision of the estimates of *L*_*V*_ and *N*_*V*_ in each case was evaluated by estimating the coefficient of error (CE) as described for systematic random samples (Cruz-Orive, 1999). By counting, on average, 197 intersections and 63 MG cells per side, mean CEs were kept at reasonably low levels (8.6 and 13.3%, respectively).

### Quantitative Analysis of Microglia on Immunofluorescent Sections: Cell Numbers

Confocal images were obtained at high resolution (2048 pixels × 2048 pixels) using the *z*-stack function of a Leica SP5 confocal microscope at 40× with a PlanApo oil-immersion objective. Five serial histological sections were selected for analysis, the rostralmost one being located just caudal to the obex. Two images were obtained per side and section that were centered on laminae I–II, often including a minor superficial part of lamina III of Sp5C. Mean tissue depth of each section was estimated from the separation between the top and bottom optical sections showing fluorescent profiles. Stacks measuring 183 × 183 × 10 μm composed of 0.5 μm-thick confocal sections were taken from 10 separate spots on each side. The density of MG cell bodies was estimated applying optical disector criteria (Sterio, 1984) on these stacks.

### Quantitative Analysis of Microglia on Immunofluorescent Sections: Length of Processes

Immunofluorescent elongated tissue components, such as filaments, axons, and dendrites, capillaries, are not good candidates for length assessments using virtual planes. Accurate detection of intersects between target structures and test lines takes time, which may enable significant photo-bleaching to occur particularly when the tissue is epi-illuminated through high-power lenses under standard fluorescence microscopy. To our knowledge, isotropic virtual planes are not implemented for confocal microscopy.

An alternative procedure was developed for this study, which is based on the application of so-called “total vertical projections” (TVPs, Cruz-Orive and Howard, 1991) to stacks of confocal images. This method allows to estimate the total length of a finite, bounded curve in 3D by counting the intersections of a set of cycloids on several flat projections of the curve obtained by rotating it about a fixed (“vertical”) axis, parallel to the projection planes. The length estimator (Eq. 1 in Cruz-Orive and Howard, 1991) is:

$$\hat{L}=2\cdot \frac{a}{l}\cdot \frac{1}{M}\,\frac{\sum_{j=1}^{n}I_{j}}{n}$$

where *a/l* is the test grid constant, representing the area of the test grid that corresponds to each cycloid arc length, *M* is the linear magnification of the vertical projections, *I*_*j*_ is the total number of intersections counted on the *j*th vertical projection, and *n* is the total number of vertical projections used. In the present study the target curve was the set of MG cell processes contained in the same stacks used for cell counting but limiting their depth to 4 μm.

The maximum *z*-projection of confocal stacks was analyzed and processed offline using FIJI (NIH, Bethesda). Images were stacked and split using ImageJ plugins in order to obtain maximum intensity projections. Five projections from each stack were obtained by rotating it at fixed equal intervals between 0 and 180° about the vertical (*y*) axis (0, 36, 72, 108, and 144°). Rotated images were saved as TIFF files prior to stereological analysis. At least two spots of the target region were analyzed on each side in the immunofluorescent sections adjacent to those immunoreacted with DAB.

Since the estimated length of MG cell processes corresponds to an undetermined fraction of incompletely sampled cells, the absolute value of *L* would be meaningless. However, the length density (*L*_*V*_) of processes is a valuable first-order parameter that can be easily obtained from volume estimations of the target tissue included in each stack at 0° rotation and after correcting for shrinkage. These estimations were obtained by point counting (or area measurement on the tissue with ImageJ), multiplying by 4 μm, the arbitrarily fixed depth of the block. The small size of this depth tried to limit the masking or overlapping of linear structures after projection, which is the foremost limitation of the TVP method (Cruz-Orive and Howard, 1991). While a certain degree of overlap was unavoidable, this seemed to be minuscule at angles <45 and >135°; when the stack was rotated at angles closer to the *z* axis (90°) masking was more considerable, particularly when immunofluorescent structures abounded. This limitation is probably causing a degree of underestimation bias in the final estimates of length densities, which will be discussed later.

**FIGURE 1:**
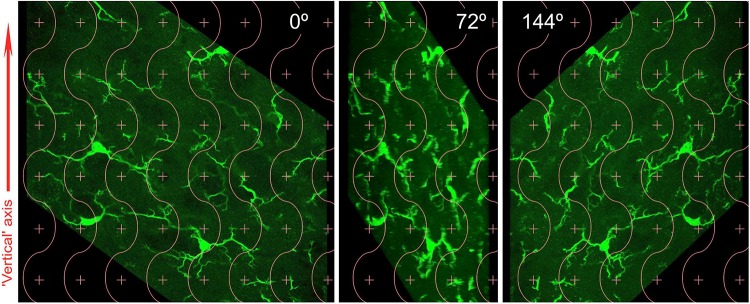
Summary view of the total vertical projections (TVP) layout (see the section “Materials and Methods” for more details). A combined point- and cycloid-grid is laid over an Iba1-immunofluorescent region of interest as it appears on a confocal 4 μm-thick stack (0°). Points are used to estimate the area of the region by point counting, which multiplied by the stack depth (4 μm) yields an estimate of the tissue volume studied. The intersections of cycloid curves with immunofluorescent processes are counted to estimate length densities. The grid is likewise projected on four more views of the region obtained by systematically rotating the stack around an arbitrary, but identifiable, “vertical” axis. Only two further images are shown to illustrate rotated views, at 72 and 144°.

Total vertical projections were processed and analyzed using Corel Draw and Photopaint (v. X3, Corel, Ottawa, ON, Canada). Image processing was limited to adjustments in brightness, contrast, and gamma to achieve optimal discrimination of fluorescent structures. An appropriate cycloid test system (Cruz-Orive and Howard, 1991; Howard and Reed, 2005) created with Corel Draw was superimposed at random positions on the vertical projections (Figure 1). Intersections of the cycloids with fluorescent processes were easy to identify when these were thin and non- or scarcely overlapping. Intersection with a thicker process was counted if it crossed the apparent central spine of the process. Under control conditions the distinction between processes and MG cell cytoplasm was unproblematic. In deafferented territory, however, some cells displayed wide pseudopodic-like extensions lacking a sharp boundary with the cytoplasm, and these were not included in the count. When two or three processes coursing at different *z* levels overlapped in the vertical projection at the crossing point of a cycloid but were identifiable as separate structures, the number of hits counted was correspondingly increased.

### Quantitative Analysis of Microglia on Immunofluorescent Sections: Cell Body Size and Number of Primary Processes

The images used for TVPs provided also a convenient material to estimate cell body size and number of primary processes in the MG cells. The vertical rotator (Jensen and Gundersen, 1993) was applied using the NewCAST software on the profiles of individual immunofluorescent MG cell bodies as shown in three evenly spaced rotated images (at 0, 72, and 144°). The mean value of the three measurements was taken as the measured value for each cell. The number of primary processes was individually registered during the application of the rotator.

### Testing for the Presence of Activated Macrophages in the Deafferented Sp5C

The presence of cells co-expressing immunofluorescence for Iba1 and CD206, or mannose receptor C type 1, was searched to investigate whether activated M2 macrophages recruited from the circulation (Gensel and Zhang, 2015) were present in the deafferented region.

An alternative procedure with the same goal was to separately identify MG and infiltrating monocytes/macrophages using flow cytometry. Tissues were placed in cold Hank balanced salt solution (HBSS) (+Ca/Mg) medium (Lonza) and mechanically dissected through a 100 μm cell strainer. Tissue suspension was centrifuged at 286 × *g* for 5 min at 4°C. Pellet was enzymatically digested in collagenase/liberase TL (2 U/ml) (Roche Diagnostics) for 1 h at 37°C. Cell suspension was filtered through a 70-μm filter with DNAse (66 U/ml) (Roche Diagnostics). Cell pellet was resuspended in 25% of density gradient and centrifuged at 521 × *g* for 20 min at 18°C. Then, leukocytes were washed and blocked with mouse Fc Block (eBioscience) prior to staining with primary antibody-conjugated fluorophores: CD45-Pacific Blue and CD11b-PE. All antibodies were commercially purchased from eBioscience. For live/dead discrimination, a fixable viability dye, carboxylic acid succinimidyl ester (CASE-AF350, Invitrogen), was diluted at 1:300 from a working stock of 0.3 mg/ml. Data were acquired on a LSRII using FACSDiva 6.0 (BD Biosciences) and analyzed using FlowJo (Treestar Inc.). No <20,000 events were recorded for each sample. Resident microglia was identified as the CD45^int^/CD11b^+^ population, whereas monocyte/macrophage were identified as CD45^high^/CD11b^+^.

### Statistics

Descriptive statistics (means and SEM) for the three parameters analyzed, *L*_*V*_, *N*_*V*_, and *L*_*N*_ were obtained from the Excel spreadsheet used to perform computations (Microsoft Office Professional Plus 2010 for Windows 10). Histograms and graphs were generated using GraphPad Prism (v. 8.0 for Windows), and eventually color-coded or reformatted using Corel X3. Statistical analyses were performed with the same software. After analyzing for normality and homogeneity of variances, comparisons between groups were made using Student’s *t*-test for the number of primary processes and mean body size and non-parametric tests for the remaining variables. The Wilcoxon signed rank test was applied for pairwise comparisons between sides and groups, and unpaired samples were compared with the Mann–Whitney *U* test. The distribution of cell body sizes in different sides and groups was compared with the two-sample Kolmogorov–Smirnov *D* test, pooling the values from all animals of the corresponding side and group. For the rest of the analyses each brain stem side was considered the sampling unit. The level of significance was set at *P*-value < 0.05, and represented on the graphs by ^∗^(*p* < 0.05), ^∗∗^(*p* < 0.01), and ^∗∗∗^(*p* < 0.001).

## Results

**FIGURE 2:**
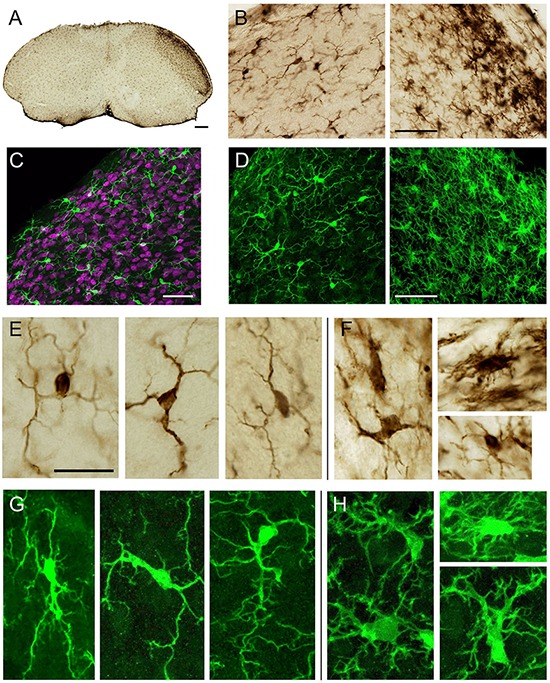
Iba1 immunolabeling of microglia (MG) in Sp5C on cross-sections of the lower brain stem in mice at different magnifications in controls and 1 week after unilateral infraorbital nerve (IoN) transection. Serial sections were reacted either for diaminobenzidine (DAB)-based immunocytochemistry and conventional light microscopy **(A,B,E,F)** or for immunofluorescence and confocal microscopy **(C,D,G,H)**. **(A)** Accumulation of immunolabeled MG on the deafferented (right) Sp5C, most pronounced in laminae I–II. **(B)** Higher-power detail of MG on the ipsi- (right) and contralateral side to the IoN transection of case shown in panel **(A)**. The right side shows higher density of immunolabeled cell bodies with heterogeneous sizes and shapes and more profuse ramifications. **(C)** Iba1-NeuN staining in a 40-μm-thick stack in a control case. The density of Iba1-immunolabeled MG was essentially uniform across laminae. **(D)** Immunofluorescent MG from 4-μm-thick stacks show similarities in cell number and laminar distribution to DAB-reacted MG **(B)** at comparable locations. They appear more extensively ramified, however, which is only partly true (see the section “Discussion”). **(E)** Representative profiles of “resting” MG in laminae I (left) and II (middle,right) at high power. **(F)** In the deafferented region, “activated” MG adopts more bizarre shapes, which include thicker protoplasmic expansions, ramifies profusely, and displays a larger range of soma size than controls. **(G,H)** Under confocal microscopy, typical immunofluorescent MG resembles DAB-reacted MG in the same laminae as controls **(E,G)** and deafferented side **(F,H)**, while appearing to be more richly ramified. Scale bars, 200 **(A)**, 50 **(B–D)**, and 20 μm **(E–H)**.

Microglia cells are distinctly stained by Iba1 immunocytochemistry or immunofluorescence in the mouse brain stem (Figure 2). They distribute fairly homogeneously with low density across the whole Sp5C, displaying small cell bodies and a few slender primary branches that radiate in all directions, except for those in laminae I or outer part of lamina II, which tend to be oriented parallel to the dorsal surface of the nucleus. The processes branch out to various degrees, and each branch may be frequently decorated with very thin and usually short processes. One week after unilateral transection of the IoN, the immunoreactivity for Iba1 increased substantially, reflecting both an increase in the density of MG cell bodies and an expansion in the density of immunolabeled processes (Figures 2A–D). The area of microgliosis extended from the rostral end of Sp5C caudalwards to end abruptly at the level of spinal segment C_1_. The “activation” of MG was most prominent in laminae I–II, decreased in laminae III–IV, and was essentially absent from the deeper laminae. Medio-laterally, it covered the lateral 4/5 of the nucleus, which somatotopically barely exceeds the target region for IoN afferents toward adjacent terminal territories of the ophthalmic (ventrolaterally) and mandibular (dorsomedial) trigeminal branches (Hayashi, 1982; Jacquin et al., 1986; Panneton et al., 2017; Fernández-Montoya et al., 2018).

### Measurable Density of Microglia Cell Bodies and Length of Processes May Depend on the Immunolabeling Procedure

**FIGURE 3:**
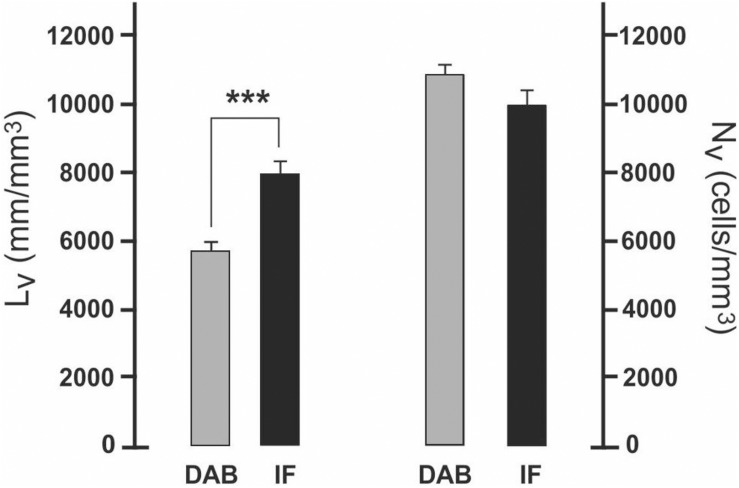
Effect on measurements of different immunostaining procedures tested on non-deafferented Sp5C. Length density of microglia (MG) processes is significantly greater (two-tailed paired *t*-test, ***p < 0.001) in immunofluorescent material (IF) than in diaminobenzidine (DAB)-reacted immunocytochemical treated parallel sections. In the same sections, minor differences between numerical densities of MG cell bodies are not significant. Although data refer to laminae I–II, occasional analyses of lamina III yielded comparable results.

The effect of different immunostaining procedures was tested stereologically by comparing data obtained on non-deafferented Sp5C. On average, numerical density of MG cell bodies showed a negligible 3% difference between procedures. However, the length density of MG processes was a highly significant 30% greater (two-tailed paired *t*-test, *p* < 0.001) in immunofluorescent than in DAB-reacted immunocytochemical treated parallel sections (Table 1 and Figure 3). The same pattern was observed between treatments on the deafferented Sp5C (4%, N.S., and 26%, *p* < 0.001, respectively). Accordingly, the estimated average total length of processes per cell varied as well (Table 1).

**TABLE 1:** Length density of microglia (MG) processes, numerical density of MG cell bodies, and length of processes per cell (mean ± SEM), sorted by side, immunoreaction type [diaminobenzidine (DAB) or immunofluorescence], and case group [controls or infraorbital nerve (IoN)-transected].

**Group**	**Iba1**	**Side**	***L*_*V*_ (mm/mm^3^)**	***N*_*V*_ (cells/mm^3^)**	***L*_*N*_ (mm/cell)**
C	DAB	L	5779 ± 348	10,311 ± 107	561 ± 34
		R	5708 ± 209	10,999 ± 418	538 ± 35
	IF	L	8427 ± 665	10,696 ± 679	825 ± 29
		R	7968 ± 560	9922 ± 576	809 ± 24
IoN	DAB	L	5615 ± 564	11,069 ± 524	525 ± 82
		R	14,128 ± 209*	49,263 ± 2397*	279 ± 25*
	IF	L	7632 ± 764	9652 ± 869	805 ± 27
		R	18,946 ± 534*	53,584 ± 2279*	361 ± 16*

Differences between sides were significant neither between the left and right sides in controls, nor between the non-deafferented side (left) in the IoN-transected animals and either side in controls. Values for the IoN-deafferented side (right) diverged consistently with respect to the other side in controls or deafferented animals (*: two-tailed *p*-values ranging from 0.023 to 0.030).

### Digital Three-Dimensional Reconstruction of Individual Microglia Cells as an Alternative for Process Length Estimation

**FIGURE 4:**
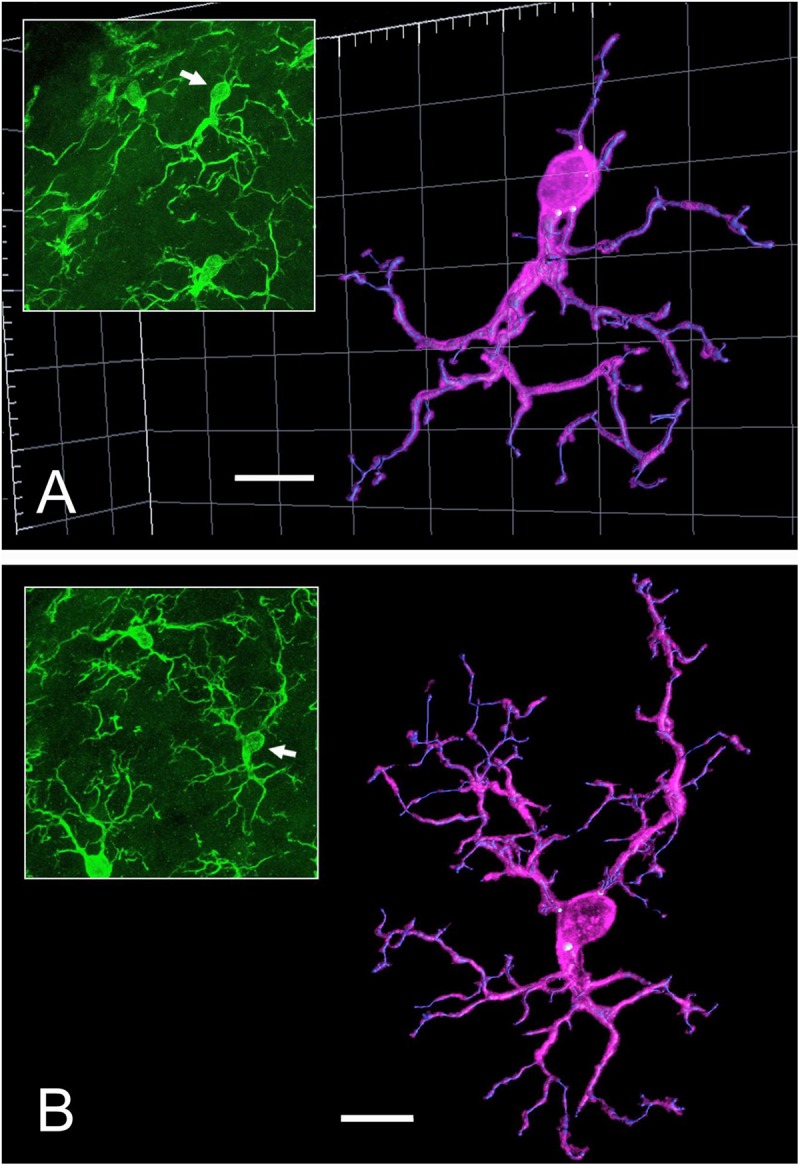
Examples of 3D reconstructions with Imaris of representative Iba1-immunostained microglia (MG) cells from two control cases. **(A)** Reconstruction of a moderately ramified cell (*L*_*N*_ = 423 μm); the view is slightly tilted with respect to its appearance in the tissue section (inset, arrow). **(B)** Example of a cell with one of the highest length of processes (*L*_*N*_ = 736 μm) among the sample studied. Scale bars, 10 μm.

The length of MG processes measured on Imaris’ digital images in two control cases (Figure 4) showed no differences between sides (592 ± 33 vs. 599 ± 31 μm, right vs. left). However, despite coming from likewise immunolabeled material, these figures were a significant 27% lower than those from the population data estimated on immunofluorescent sections stereologically analyzed with virtual planes (Table 1; see the section “Discussion”).

### Deafferentation by Infraorbital Nerve Transection Causes a Discordant Ipsilateral Increase in Cell Number and Process Length

**FIGURE 5:**
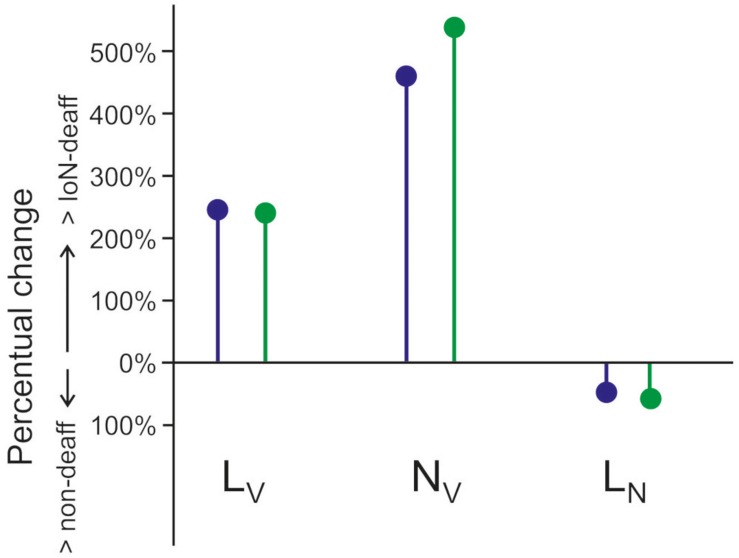
Effect of infraorbital nerve (IoN) transection on process length (*L*_*V*_), numerical densities of cells (*N*_*V*_), and length of processes per microglia (MG) cell (*L*_*N*_). Values for the deafferented side are compared to pooled values from non-deafferented side and controls and expressed as overall mean percentages. Purple, data from diaminobenzidine (DAB)-reacted material; green, data from immunofluorescent tissue.

Iba1 expression in deafferented laminae I–II displayed the most marked changes. The magnitude of these changes differed, however, for cell bodies’ and process length densities. MG cell bodies increased between 445 (in DAB-reacted immunocytochemical material) and 555% (in immunofluorescent material). Density of immunolabeled processes increased just by about 250% in both types of labeling (Table 1). No significant differences were found in either parameter between control mice and the side contralateral to the deafferentation in IoN-transected animals. This discrepancy resulted in a significant reduction of mean length of process per cell in the deafferented side to 53 or 45%, in DAB-reacted or immunofluorescent tissue, respectively (Figure 5).

### Deafferentation-Induced Microgliosis Entails Marked Changes in Cell Body Shape and Size

The appearance of MG in control cases (and contralaterally to the deafferented side in transected cases) reproduced faithfully classical descriptions of “resting” or “surveillant” MG in the brain stem and elsewhere (review in Kettenmann et al., 2011): from ovoid, fusiform, or triangular, predominantly small cell bodies emerge three to six primary branches that divide into second- and third-order, less frequently into higher order branches. Thinner, filopodia-like processes commonly appear anywhere along the branches as well as occasional bulbous swellings that, near the soma, can reach considerable size (Figures 2E,G).

**FIGURE 6:**
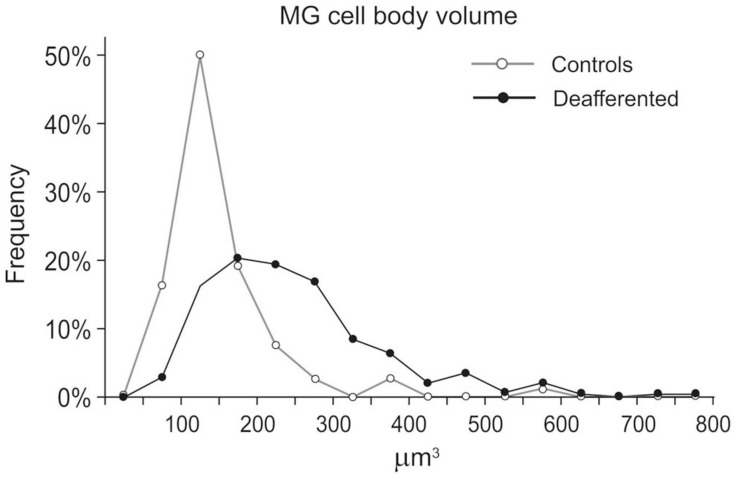
Distribution of microglia (MG) according to cell body size, grouped into 16 classes, between 50 and 800 μm^3^ at 50 μm^3^ step increments. Values from MG in three controls (*n* = 110) were pooled as were values from MG in three deafferented Sp5C (*n* = 283). MG cell body size was on average 67% larger in the deafferented region (249 ± 118 vs. 149 ± 74 μm^3^, mean ± sd). Cell size distribution differed significantly between groups (*p* < 0.001, Kolmogorov–Smirnov *D*-test).

In the deafferented region, the “activated” MG display a variety of shapes, from small cell bodies with few primary process (a minor proportion) to larger cells (Figure 6) with prominent cytoplasmic expansions and abundant branches, usually shorter and without any preferential orientation (Figures 2F,H). Not including in the count thick cytoplasmic outgrowths, the number of primary branches more than duplicated those in controls from stereology-based material (9.0 ± 0.2 vs. 4.1 ± 0.1, *p* < 0.001) and Imaris’ reconstructions (4.0 ± 0.2). Process-free, “ameboid” type profiles were very seldom found.

### Iba1-Expressing Cells in the Deafferented Sp5C Correspond to Microglia, Not Infiltrating Macrophages

**FIGURE 7:**
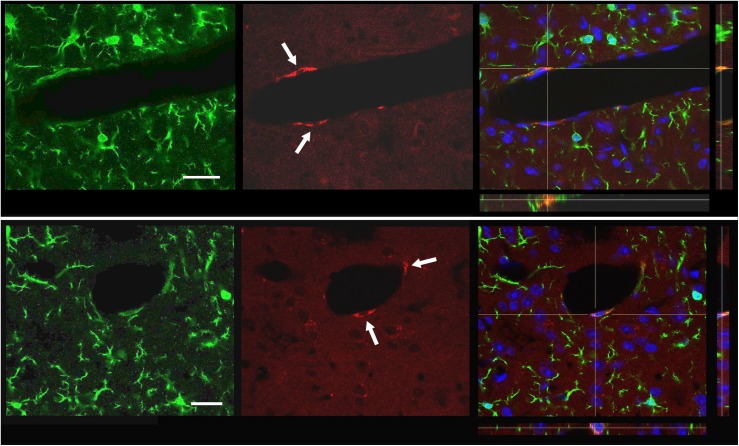
Co-localization of Iba1 (green) and CD206 (red) in putative perivascular macrophages in close apposition to the abluminal surface of small blood vessels (arrows), above and below a blood vessel. The merged picture and orthogonal XZ and YZ projections on the right include bisbenzimide nuclear staining (blue). Scale bars, 20 μm.

**FIGURE 8:**
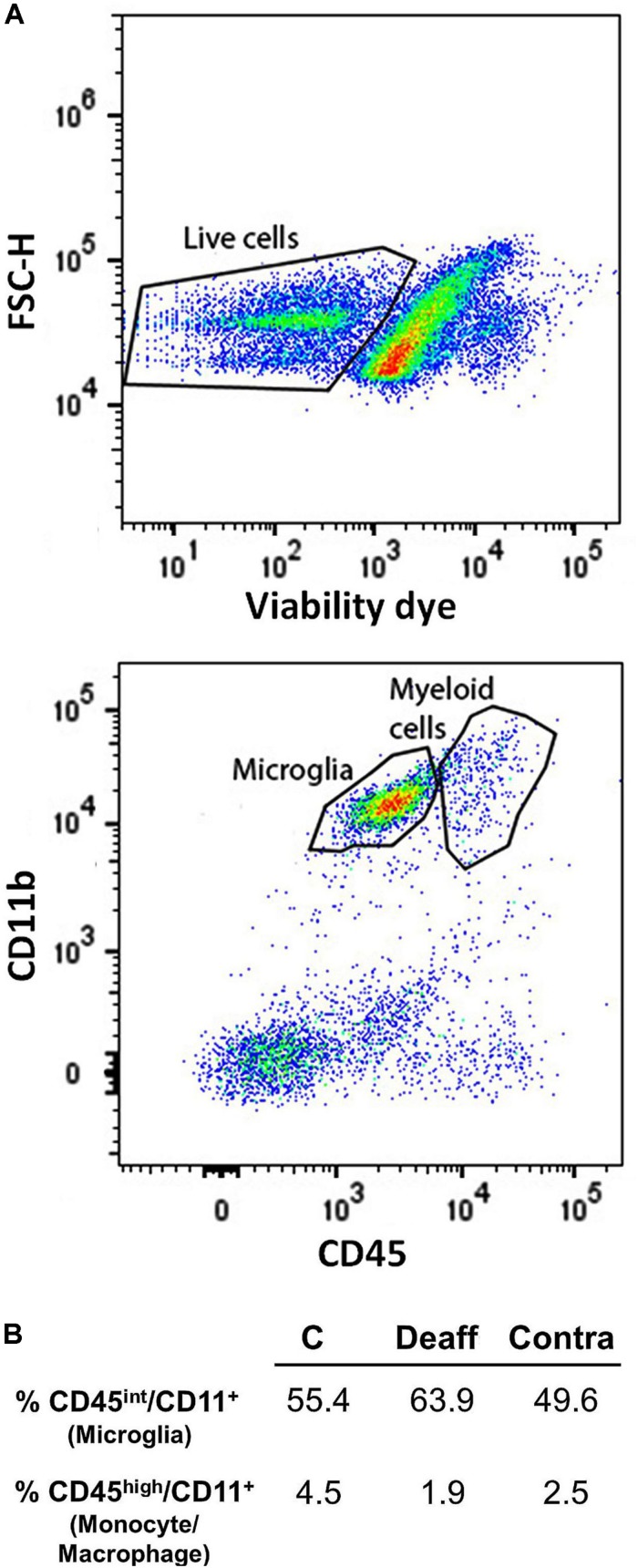
**(A)** Representative gating strategy for flow cytometric analysis in the brain stem. Singlets were gated on FSC-A *versus* FSC-H and live cells were gated (top). CD45^int^ (microglia) and CD45^high^ immune cells were gated based on CD45 staining *versus* CD11b, a cell surface antibody used to identify myeloid cells (bottom). **(B)** Percentage of positive cells (microglia CD45^int^/CD11b^+^ and monocyte/macrophage CD45^high^/CD11b^+^) in control, and deafferented animals. Values represent percentage of positive cells from pooled caudal spinal trigeminal nuclei of four (controls, bilateral) and six animals (ipsi- and contralateral to deafferentation).

A limited number of cellular profiles that were strongly labeled for CD206 appeared on both sides of the deafferented cases. All of them co-expressed Iba1 and were always located in close apposition to the abluminal surface of small blood vessels, which suggested they could correspond to a subset of perivascular macrophages (Lapenna et al., 2018). None of the more intensely Iba1^+^ parenchymal cells with typical MG shapes were CD206^+^ either in deafferented laminae or elsewhere (Figure 7). Consistently, our FACS analysis revealed that only a minor fraction of cells expressing CD11b, a common marker of myeloid lineage cells, also expressed a high level of CD45, which, together, point to monocyte/macrophage cell types. These levels were essentially the same in controls and deafferented cases (Figure 8).

## Discussion

In this study we describe the fundamental morphometric parameters of Iba1-immunolabeled MG in the caudal division of the trigeminal nuclear complex in control mice and at the peak of the microgliosis induced by deafferentation due to unilateral IoN transection. All parameters are estimated by different image analysis and stereological methods suited to the immunostaining procedure employed (DAB-reacted, transmitted light microscopy, or immunofluorescence and confocal microscopy) and the type of target structure aimed at (either single MG cells or population data in a given region). Measurements are assumption-free concerning structures’ size, shape, or orientation, and represent 3D values. Overall, MG cell density in the superficial laminae of the deafferented Sp5C increased five times while density of MG processes increased 2.5 times, revealing a net 50% decrease in the mean length of processes per cell. MG cell bodies’ size and shape and initial branching pattern showed only moderate variability in controls and contralaterally to the IoN-transection. On the deafferented side, however, MG displayed on average larger somata and remarkable diversity in shape. While we cannot discard that some MG may have proliferated outside and then migrated into the area of microgliosis, previous studies showing the temporal course of MG proliferation in deafferented areas as well as the similarity between MG elsewhere in the ipsi- and contralateral sides in our study indicate that both proliferation and migration are mostly local phenomena within the deafferented area. We also provide evidence that the vast majority of the Iba1-expressing cells counted are MG since no infiltrating macrophages appear to be present in the control or deafferented nucleus, and the relatively rare Iba1- and CD206-expressing cells that differ in shape, are attached to blood vessels and lack processes.

### The Unsolved Challenge of Microglia Morphometry

The interest in understanding how the plastic and dynamic MG structure informs on the variegated roles that these cells play in health and pathology has not ceased to grow in the last years. Accordingly, morphometric approaches keep evolving in an attempt to overcome the multiple difficulties encountered when trying to extract accurate quantitative data from such a complex structure with a reasonable efficiency (Beynon and Walker, 2012; Kozlowski and Weimer, 2012; Plog et al., 2014; Torres-Platas et al., 2014; Young and Morrison, 2018). Most current methods include segmentation (mainly focused on thresholding) and skeletonization of digital images and manual or semiautomatic tracing and reconstruction of immunolabeled cells. These methods target, ideally, individual and completely stained cells. For *ex vivo* models, such as hippocampal slices or whole-mount retinal preparations, time-lapse studies provide excellent approaches to quantify individual cell motility and process elongation/retraction (Stence et al., 2001; Lee et al., 2008; Damani et al., 2011). Design-based stereology, so far, has been limited to cell counting (Beggs and Salter, 2007; Wu et al., 2014; Perkins et al., 2018) and more recently, has been combined under certain favorable conditions with digital cell tracing (Papageorgiou et al., 2015) and advanced automatic segmentation algorithms (Ahmady Phoulady et al., 2019).

None of these approaches, however, is able to handle satisfactorily all the difficulties posed by MG morphometry even if the first condition for consistency and reliability of the data, high quality histological processing, and microscopy, is met. Briefly outlined, single cell reconstructions are hard or impossible to obtain from densely packed MG and risk serious biases when cell processes are not entirely contained in the reconstruction, or when supervised or unsupervised tracing erroneously assigns processes to a given cell. Most often MG processes are measured from tissue sections thin enough to guarantee complete penetration of the immunolabeling but which cannot ensure that the whole set of processes is included in the section. From intracellularly filled and presumably fully labeled MG in very thick (up to 200 μm) slices, the mean linear span of relatively isotropic cells reaches at least 60–90 μm in different brain regions (Hayashi et al., 2013; De Biase et al., 2017; Leon-Espinosa et al., 2018). Moreover, sampling should be bias-free, lest the largest and most conspicuous cells had a disproportionately higher probability of being sampled.

Our design-based stereological approaches provide unbiased sampling of both cell bodies and processes. Separate stereological estimations of process length densities, which have not been attempted before, have been tailored here for DAB-reacted and immunofluorescent material. Yet, some words of caution are in order: (1) Even using the highest possible magnification, DAB-based and light-transmitted material analyzed with virtual planes yielded 30% lower measures of process length than immunofluorescent sections analyzed with TVP, a difference that could be attributed to the lower sensitivity of the DAB signal for very thin processes and/or the higher capability of a laser source to excite even small amounts of fluorescent molecules. A much larger difference (58%) recently reported by Young and Morrison (2018) applying an ImageJ plugin to measure binary and skeletonized confocal images was mainly due to a marked reduction of DAB-based values. (2) Compared to Imaris-based digital reconstructions of control MG, TVP yielded 27% higher length values, which could probably be attributed to an incompleteness of the cell arbors reconstructed by Imaris. (3) The number of intersections of cycloids with labeled processes using TVP fell on sections rotated >45° due to overlapping, which could lead to an underestimation of length densities. This was a minor problem in control material but could be substantial in zones with heavy microgliosis. (4) While TVP can be easily applied with no need for special equipment (aside from good quality confocal microscopy), virtual planes and optical rotator require a stereological setup that provides the test grids and software to perform the computations needed for the corresponding estimators. (5) With a limited amount of hands-on training, nevertheless, results are rewarding: independent analysis by two authors (CA and NG-M) of the same sections on two cases, one control and one IoN-transected, yielded better than 97% agreement on estimation of cell numbers and process length densities in controls, and better than 89% agreement on process length densities in the deafferented Sp5C. Results were similar in DAB-based and immunofluorescent materials.

### On the Spatial and Temporal Features of Deafferentation-Induced Microgliosis

Following peripheral nerve injury, microgliosis, as defined by an increase in cell number, enlargement of cell body, and thickening of processes, is already apparent in the deafferented spinal or brain stem territory (Eriksson et al., 1993; Melzer et al., 1997; Coyle, 1998) by 4 days postlesion and reaches a maximum by 7 days postlesion. In the spinal dorsal horn following transection of a lumbar spinal nerve the MG enter promptly into a proliferative burst that takes place within a 24–96 h time window, peaking at 48–60 h postlesion (Liu et al., 2000; Gu et al., 2016; Kohno et al., 2018). This MG reaction is not restricted to deafferentations due to loss of peripheral afferents. Early studies showed a similar proliferative pattern in the hippocampal formation following partial deafferentations by lesion of the perforant path or the commissural projections (Avendaño and Cowan, 1979; Gall et al., 1979; Avendaño, 1983). Moreover, while at early stages proliferating MG appear beyond the deafferented region, past the proliferative burst MG migrate to concentrate in the directly deafferented territory and closely adjacent zones (Gall et al., 1979; Beggs and Salter, 2007; present results). The obvious preference of microgliosis for the superficial laminae I–II may reveal an additive effect in these laminae of a dense distribution of nociception-related thin unmyelinated afferents (Fernández-Montoya et al., 2018) and, given the greater sensitivity of small ganglion neurons in the trigeminal ganglion to peripheral axotomy (Lagares et al., 2007), a putatively larger fraction of fibers in the course of an irreversible degeneration.

### Blood-Borne Monocytes Do Not Contribute to Nerve-Injury Induced Microgliosis in Sp5C

The coexistence of activated resident MG and infiltrating blood-borne macrophages is a common finding in inflammatory and degenerative brain and spinal disorders, and efforts are being directed to distinguish between those cell types and their varying molecular and genetic expression under different homeostatic and pathologic conditions (for reviews, see Benakis et al., 2015; Chu et al., 2018; David et al., 2018). If such coexistence were to occur as well within deafferentation-induced microgliosis, a view favored by some authors (Echeverry et al., 2011), it would demand clarifying the contribution of each cell type to whatever morphometric data were collected. However, more recent data using bone marrow chimeric mice and double transgenic mice that allowed specific staining of each cell type, strongly support the absence of significant infiltration of circulating monocytes in the peripheral nerve-dependent microgliosis (Gu et al., 2016; Tashima et al., 2016). This is also supported by our failure to find Iba1-immunolabeled cells coexpressing CD206, a well established marker of M2 macrophages (Gensel and Zhang, 2015), aside from blood vessels-attached putative perivascular macrophages as well as the results obtained by FACS, where we observed no differences of CD45^high^/CD11b^+^ cells in controls and deafferented animals. Therefore, we may conclude that infiltrating macrophages do not contribute to local microgliosis, at least during the first 2 weeks after nerve injury.

### The Methods Proposed Here Are Applicable to Microglia Elsewhere: A Novel Proposal for the Morphometry of Microglia

The decisive involvement of MG in the pathogenesis of trigeminal neuropathic pain (Tsuda et al., 2005; Suter et al., 2009; Calvo and Bennett, 2012; Daigo et al., 2012; Gu et al., 2016) was what drove this study in the first place. In addition, the difficulties emerging to quantitatively assess the morphological changes of MG in different models led us to focus the study on the model that more severely denervated Sp5C and elicited prominent microgliosis. Our approach applies relatively easily to low-density MG populations but also works, with limited biases and a notable degree of precision and efficiency, in territories where MG cell bodies and processes form dense meshworks. In sum, we believe that it is possible to extend the procedures delineated here to any morphometric population analysis of MG under different physiological or pathological conditions.

## Data Availability Statement

The datasets generated for this study are available on request to the corresponding author.

## Ethics Statement

The animal study was reviewed and approved by the Ethical Committee of the Autonomous University of Madrid, in accordance with European Community’s Council Directive 2010/63/UE.

## Author Contributions

CA and NG-M contributed the initial biological idea for the study and performed the stereology and image analysis. NG-M and YM carried out most experimental procedures. NG-M was responsible for histological data acquisition, and corresponding results were analyzed by NG-M, CA, and PN. AP-A and JE carried out and analyzed the FACS study. CA, NG-M, and PN were mainly responsible for writing the article. All authors participated in the discussion and approved the final manuscript.

## Conflict of Interest

The authors declare that the research was conducted in the absence of any commercial or financial relationships that could be construed as a potential conflict of interest.
